# Genotypic characterization directly applied to sputum improves the detection of *Mycobacterium africanum* West African 1, under-represented in positive cultures

**DOI:** 10.1371/journal.pntd.0005900

**Published:** 2017-09-01

**Authors:** C. N’Dira Sanoussi, Dissou Affolabi, Leen Rigouts, Séverin Anagonou, Bouke de Jong

**Affiliations:** 1 Mycobacteriology Unit, Institute of Tropical Medicine, Antwerp, Belgium; 2 Laboratoire de Référence des Mycobactéries, Centre National Hospitalier Universitaire de Pneumo-Phtisiologie de Cotonou, National Tuberculosis Programme, Cotonou, Benin; 3 Department of Biomedical Sciences, University of Antwerp, Antwerp, Belgium; Institut Pasteur, FRANCE

## Abstract

**Background:**

This study aimed to compare the prevalence of *Mycobacterium tuberculosis* complex (MTBc) lineages between direct genotyping (on sputum) and indirect genotyping (on culture), to characterize potential culture bias against difficult growers.

**Methodology/Principal findings:**

Smear-positive sputa from consecutive new tuberculosis patients diagnosed in Cotonou, (Benin) were included, before patients had started treatment. An aliquot of decontaminated sputum was used for direct spoligotyping, and another aliquot was cultured on Löwenstein Jensen (LJ) medium (90 days), for indirect spoligotyping. After DNA extraction, spoligotyping was done according to the standard method for all specimens, and patterns obtained from sputa were compared versus those from the derived culture isolates. From 199 patient’s sputa, 146 (73.4%) yielded a positive culture. In total, direct spoligotyping yielded a pattern in 98.5% (196/199) of the specimens, versus 73.4% (146/199) for indirect spoligotyping on cultures. There was good agreement between sputum- and isolate derived patterns: 94.4% (135/143) at spoligotype level and 96.5% (138/143) at (sub)lineage level. Two of the 8 pairs with discrepant pattern were suggestive of mixed infection in sputum. Ancestral lineages (Lineage 1, and *M*. *africanum* Lineages 5 and 6) were less likely to grow in culture (OR = 0.30, 95%CI (0.14 to 0.64), p = 0.0016); especially Lineage 5 (OR = 0.37 95%CI (0.17 to 0.79), p = 0.010). Among modern lineages, Lineage 4 was over-represented in positive-culture specimens (OR = 3.01, 95%CI (1.4 to 6.51), p = 0.005).

**Conclusions/ Significance:**

Ancestral lineages, especially *M*. *africanum* West African 1 (Lineage 5), are less likely to grow in culture relative to modern lineages, especially *M*. *tuberculosis* Euro-American (Lineage 4). Direct spoligotyping on smear positive sputum is effective and efficient compared to indirect spoligotyping of cultures. It allows for a more accurate unbiased determination of the population structure of the *M*. *tuberculosis* complex.

**Trial registration:**

ClinicalTrials.gov NCT02744469

## Author summary

The vast majority (95%) of tuberculosis (TB) patients worldwide live in low-income countries, including in West-Africa. Typing the bacteria responsible for TB (tuberculosis; *Mycobacterium tuberculosis* complex) is important for targeted TB control. Typing is usually performed on isolates obtained after the culture isolation of TB bacteria in the sputa from patients. However, cultures can be false negative, and some ‘ancestral’ strains, only found in West-Africa (*Mycobacterium africanum*), require more time (90 days versus the usual 56 days) to grow in culture. To characterize potential culture bias against such “difficult growers”, we compared the performance of direct typing (on sputum) relative to its yield on culture isolates. We found that ancestral types of TB bacteria were significantly less likely to grow in culture despite the 90-day incubation. This suggests that typing results of cultured isolates are not representative of the diversity in the population of TB bacteria causing disease in patients. Typing sputum directly is effective and can be used for a more precise, unbiased determination of the proportion of different TB bacteria in a population.

## Introduction

Tuberculosis (TB), caused by bacteria of the *Mycobacterium tuberculosis* complex (MTBc), remains a public health problem. Globally, over 8 million new patients with TB disease arise each year, including 2 million deaths. The vast majority (95%) of global TB is detected in limited-resource countries [1], including West-Africa. Each year in Benin, over 4000 cases of TB are detected, and the incidence of smear-positive pulmonary TB is 39 per 100000 inhabitants.

Genotypic characterization is important in order to understand the population structure of the MTBc for better insights into endemic- and epidemic strains and to identify instances of nosocomial transmission or laboratory contamination. *M*. *tuberculosis sensu stricto* and *M*. *africanum* sub-species within the MTBc have been subdivided into 7 main lineages of human importance [2,3]. These 7 MTBc lineages are classified as ancestral (or ‘ancient’) (Lineages 1, 5, 6) [4,5], intermediate (Lineage 7) [3,4] and modern lineages (Lineages 2,3,4) [4]. Lineage 5 (*M*. *africanum* West African 1) and Lineage 6 (*M*. *africanum* West African 2) are only found in West- and Central Africa, where they cause up to 40% of all TB [6,7]. Recent reports suggested a decrease in prevalence of *M*. *africanum* in some West-African countries [8–10]. Whether methodological issues explain the apparent disappearance of *M*. *africanum* has not been excluded to date.

For the determination of the population structure of the MTBc, genotyping is usually applied on culture isolates [11]. *M*. *africanum* grows significantly slower than the other members of the MTBc (*M*. *tuberculosis sensu stricto*) [12] and cultures should be incubated for 90 days rather than the usual 56 days, before reporting a negative result [13]. However, even this extended incubation time may not permit recovery of *M*. *africanum* isolates at the same rate as *M*. *tuberculosis*, and thus bias the population structure derived from cultured isolates, especially in settings where *M*. *africanum* is endemic. Differences in expression of genes involved in metabolism pathways of the various MTBc lineages may also affect their growth in culture, as recently reported for *M*. *africanum* Lineage 6 which has an under-expression for the gene (*Dos R*) involved in adaptation to lower oxygen tension relative to Lineage 4 [14]. For isolation, of some MTBc species, including *M*. *africanum*, the need for pyruvate to support growth in culture [15] has been known for a long time [16].

Few studies evaluated genotyping, such as spoligotyping, directly on clinical specimens such as sputa [17,18], sputum smears [19], paraffin wax-embedded tissues [20] or mummified remains of human [20]. Only one study from Brazil, where *M*. *africanum* is not endemic, compared spoligotyping on sputum to spoligotyping from the respective isolates [21]. Moreover, to the best of our knowledge, no study has investigated whether the proportional prevalence of MTBc lineages differs among specimens with a positive culture versus culture-negative specimens.

In this study, we determined the performance of spoligotyping on sputum (‘direct spoligotying’) relative to its yield on culture (‘indirect spoligotyping’) for genotypic characterization of MTBc, and evaluated for a potential culture bias against difficult-growers, even when incubation was prolonged to enhance detection of *M*. *africanum*.

## Methods

### Ethics statement

This study is part of the BeniDiT study that has been approved by the national ethics committee of Benin, the Institutional Review Board of the Institute of Tropical Medicine of Antwerp, Belgium and the ethics committee of the University of Antwerp. It is registered on ClinicalTrials.gov under the registration number NCT02744469. All sputa were anonymized before laboratory analyses.

### Patients/Specimens and laboratory analyses

**Fig 1:**
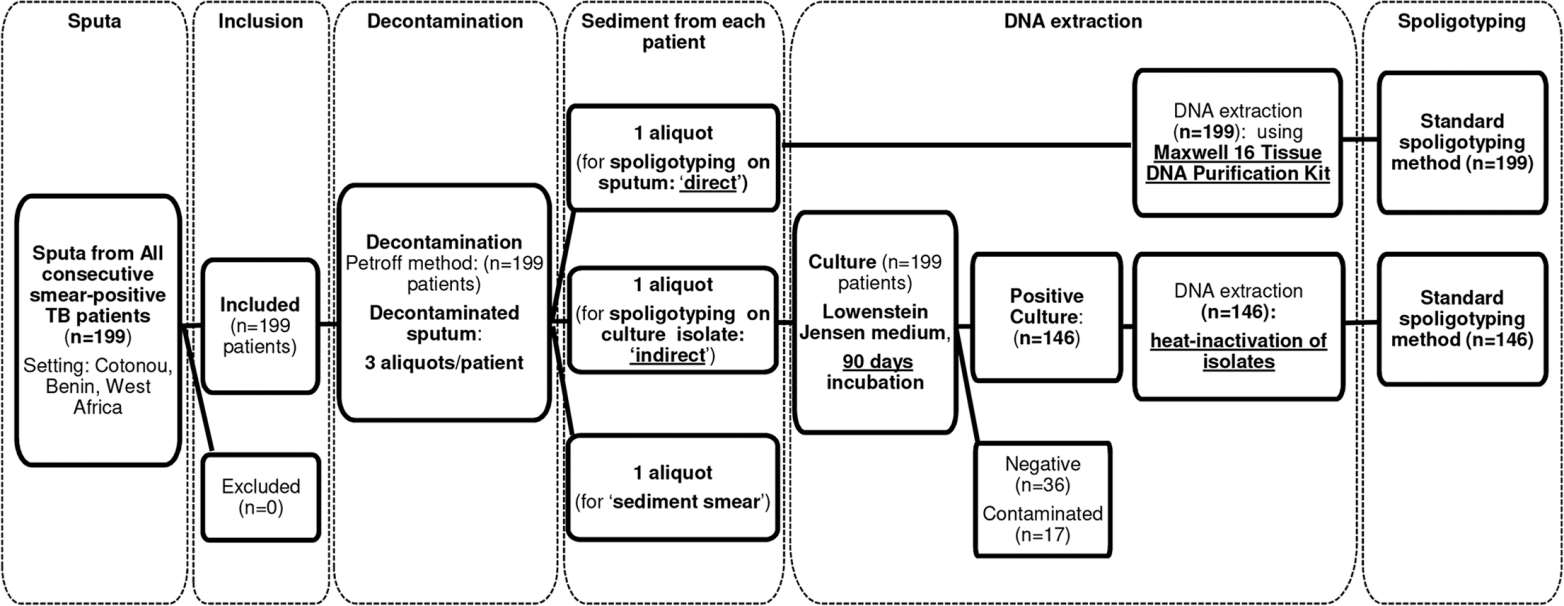
Patients, specimens flow diagram and laboratory analyses.

Smear-positive sputa from consecutive new TB patients diagnosed in the Centre National Hospitalier Universitaire de Pneumo-Phtisiologie in Cotonou, (Benin) were prospectively included (Fig 1), before patients initiated TB treatment. Laboratory analyses were conducted in the National Reference Laboratory for Mycobacteria (Laboratoire de Référence des Mycobactéries) in Cotonou, Benin.

#### Preparation of sediment aliquots, culture and sediment microscopy

When patients were identified as having (direct) acid-fast bacilli (AFB) positive pulmonary TB by fluorescent microscopy, their smear-positive sputum was included in the study. Each sputum was decontaminated using the Petroff method (15 minutes in an equal volume of 4% NaOH corresponding to 2% NaOH final concentation, neutralized with 1N HCl containing phenol red). Pellets obtained after centrifugation (3000 g, 4°C, 20 min) were resuspended with 2 mL phosphate-buffered saline. An aliquot of decontaminated sputum was used to prepare a smear, another aliquot was cultured on Löwenstein-Jensen (LJ, 2 slants of LJ containing 0.75% glycerol and 1 slant of LJ containing 0.5% pyruvate) and incubated for 90 days (13 weeks) at 37°C before being reported as negative, and another aliquot was used for direct spoligotyping. Slides from decontaminated sputa were auramine stained and read on a fluorescence microscope for acid-fast bacilli grading as previously described [22].

#### DNA extraction from culture isolates

If culture was positive, DNA was extracted from culture isolates by transferring a loop of bacilli into 300 μL molecular grade water, followed by heat-inactivation for 20 minutes at 100°C [23].

#### DNA extraction from decontaminated sputa (sediments)

DNA was extracted by the Promega Maxwell16 Tissue DNA purification kit AS1030 [24] after a prior heat-inactivation (at 100°C for 5 minutes) [23] of 200 μL decontaminated sputum and digestion with 50 μL of 20mg/mL proteinase K (at 62°C overnight) in 200 μL of lysis buffer (10 mM tris-HCl pH 7.5, 10 mM NaCl, 10 mM EDTA, 0.5% SDS), using the Maxwell 16 machine model AS2000 ver 4.9 (Promega) [24]. DNA was eluted in 300μL Maxwell elution buffer [24]. Positive (mycobacterial sediment known to be PCR-positive) and negative (molecular grade water) controls were included for DNA extraction from sputum.

#### Spoligotyping

Spoligotyping was done according to the standard method previously described by Kamerbeek *et al*. [25] on in-house prepared membranes for all samples (sputa and culture isolates) (Fig 1). Each PCR reaction (50μL) contained 5 μL of DNA from the sputum or culture isolate. Specimen flow and laboratory analyses are summarized in Fig 1. The reference *M*. *tuberculosis* strains H37Rv, *M*. *bovis* BCG and a negative control (molecular grade water) were included in each PCR and hybridization run.

For each patient, the spoligotype pattern from sputum was compared to the one from the respective isolate. If discrepant spacers were identified, the process (DNA extraction, amplification, hybridization for spoligotyping) was repeated from both the sputum and the isolate for confirmation.

### Lineage assignment

Spoligotype patterns were recorded in an Excel file using a binary code (1 for presence of a given spacer and 0 for the absence of a given spacer). Entered profiles were verified and validated by an independent person. The persons who typed and validated the data were blinded to the spoligotype pattern of the corresponding sputum or isolate. The Excel file was loaded into the TBlineage database http://tbinsight.cs.rpi.edu/run_tb_lineage.html [26] for lineage assignment. Sub-lineages (spoligotype families) were obtained by loading the Excel file with the spoligotype patterns in the SPOTCLUST database http://tbinsight.cs.rpi.edu/run_spotclust.html [27].

### Statistical analyses

Data was analyzed using the statistical software Stata/IC 12.0 (StataCorp). The two-group proportion test or the Fisher Exact test was used to analyze independent data. Mc Nemar Chi2 test was used to compare paired proportions. Two-sided p-values were calculated and for differences in proportion, odds ratios were calculated along with 95% confidence interval. Differences were considered statistically significant when p<0.05.

## Results

### Success of direct spoligotyping versus indirect spoligotyping

From the 199 recruited TB patients and their sputum samples, 146 (73.4%) yielded a positive culture, whereas 36 (18.1%) remained negative and 17 (8.5%) were contaminated. Spoligotype patterns were obtained for all the 146 culture isolates, and for 196 of the 199 sputa, yielding an overall success for direct spoligotyping of 98.5%. All of the extraction controls and amplification/hybridization controls yielded expected results, and repeat spoligotyping for discordant results between sputum and culture confirmed the original patterns. Stratified by culture result, direct spoligotyping reached a success of 100% (53/53) for culture-negative or contaminated sputa, and 98% (143/146) for culture-positive sputa. Microscopy was negative in 6 sediments after decontamination, while all the others had positive microscopy. Of the 6 microscopy negative sediments, 3 failed direct spoligotyping and 2 others had a negative culture.

Spoligotype patterns were available for 98.5% of sputa versus 73.4% of cultures (Table 1).

**Table 1:** Availability of spoligotype patterns depending on the spoligotyping method used.

		Direct spoligotyping		Total	D_Culture—Sputum_ (95% CI),
		Yes	No		
**Indirect spoligotyping**	**Yes**	143	3 ^a^	146 (73.4%)	• D: **-25.1%** (-32.1 to -18.1),
	**No**	53 ^b^	0	53 ^b^	
**Total**		196 (98.5%)	3 ^a^	199	

^a^ The 3 specimens with failed direct spoligotyping were culture positive and successfully typed indirectly

^b^ Culture-negative or contaminated specimens

D: Difference

### Spoligotype patterns from direct spoligotyping versus indirect spoligotyping

**Fig 2:**
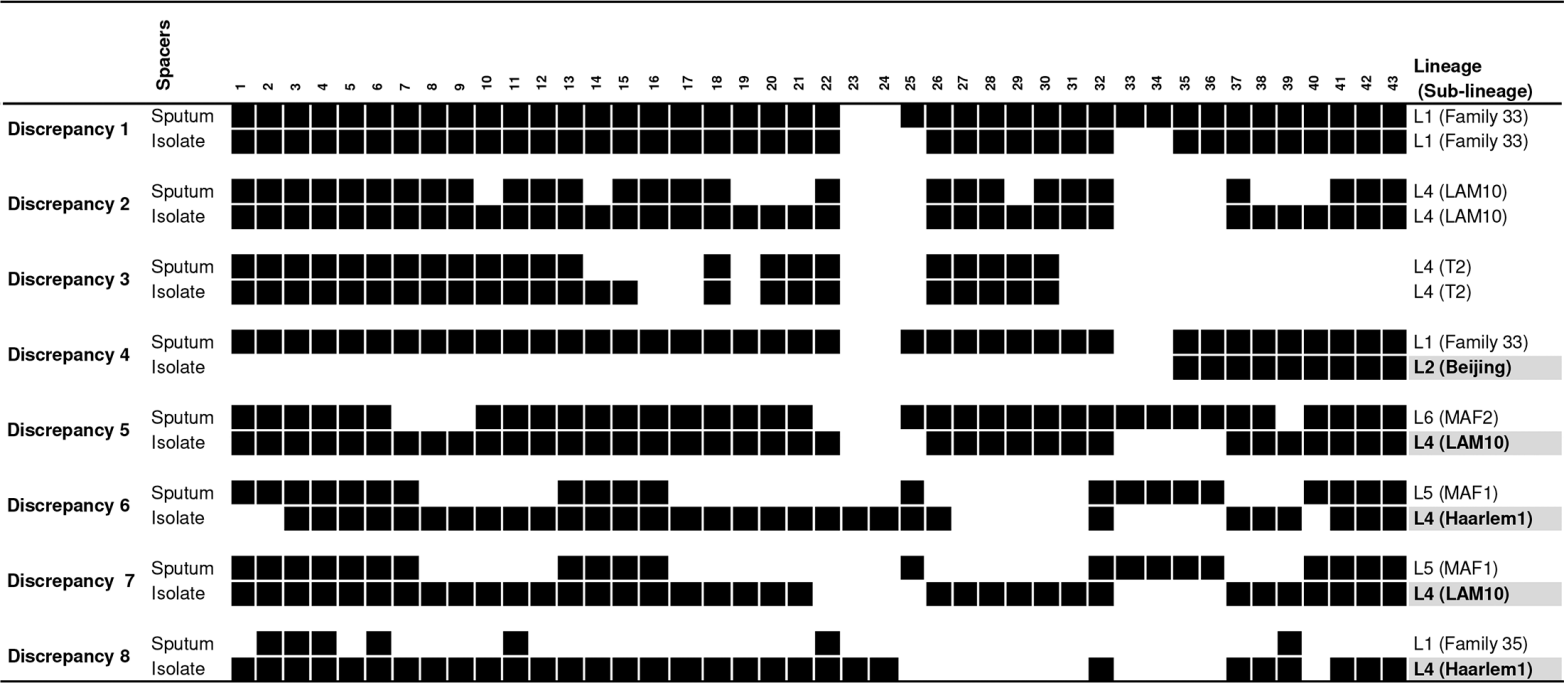
Discrepancies: Spoligotype profile, lineage and sub-lineage.

Comparison between respective direct and indirect spoligotypes showed 94.4% (135/143) agreement. In total three types of discrepancies were observed (Fig 2): mixed infection with one pattern found in sputum and the other found in the culture isolate (n = 3, discrepancy 5–7), mixed infection with overlapping spoligotype patterns in sputum (n = 2, discrepancies 1 and 4), and false negative (missing) spacers in sputum (n = 3, discrepancies 2, 3 and 8). Five (5) of these patterns led to inter-lineage discrepancies, and three (3) to intra-lineage discrepancies.

For inter-lineage discrepancies, sub(lineages) observed in isolates are shaded grey. The five inter-lineage discrepant pairs (discrepancy 4–8) showed patterns suggestive of a simultaneous presence of ancestral and modern lineages, while these yielded only the ancestral lineage in sputum and only the modern lineage in culture. Three (discrepancy 5–7) of these five inter-lineage pairs showed this (ancestral *M*. *africanum* in sputum and modern Lineage 4 in culture), without any other possible explanation, while the other two (discrepancy 4 and 8) can also be interpreted as follows. Inter-lineage pair 8 and intra-lineage pairs 2 and 3 showed patterns suggestive of false negative spacers in sputum (spacer present in isolate but absent in sputum). Intra-lineage pair 1 and inter-lineage pair 4 showed patterns suggestive of overlapping spoligotype signatures in sputum (discrepancy 1 and 4) and/or in isolate (discrepancy 1). Discrepancy 4 suggested an overlapping of Lineages 2 and 4 signatures in sputum, with only the Lineage 2 grown in culture. Discrepancy 1 was suggestive of overlapping spoligotype signatures in sputum and in culture isolate that could be a mixture of Lineages 2 and 4.

### Distribution of lineages in culture positive versus culture negative sputa

The distribution of lineages in culture-positive sputa versus directly in sputum with unsuccessful culture differed, with Lineage 5 (*M*. *africanum* West African 1) being significantly less prevalent among culture-positive sputa (OR = 0.48 95%CI (0.24 to 0.94) p = 0.033, Table 2). This association became more significant when contaminated cultures were excluded from the analysis (OR = 0.37, 95%CI (0.17 to 0.8), 21% vs 41.7%, p = 0.011, Table 2). Ancestral lineages (Lineages 1, 5 and 6) were significantly less present among culture-positive sputa (OR = 0.33, 95%CI (0.16 to 0.7), 37.1% vs 63.9%, p = 0.004, Table 2). Lineage 4 (*M*. *tuberculosis* Euro-American), a modern lineage, was most overrepresented in culture-positive sputa (OR = 2.81, 95%CI (1.30 to 6.03)55.2% vs 30.5%, p = 0.008, Table 2).

**Table 2:** Effect of prior culture on spoligotyping analysis for MTBc lineage detection.

Lineages/Groups	All patients ^a^ % (n = 199)	All sputa ^b^ % (n = 196)	Culture positive sputa ^b^ % (n = 143)	Culture Negative & Contaminated sputa				Culture Negative sputa (only)			
				% (n = 53)	Odds ratio (Odd_Pos cult_ / Odd_Neg & Cont cult_) with 95% CI	% Difference (P_Pos cult_—P_Neg & Cont cult_) with 95% CI	p-value ^c^	% (n = 36)	Odds ratio (Odd_Pos cult_ / Odd_Neg cult_) with 95% CI	% Difference (P_Pos cult_ -P_Neg cult_) with 95% CI	p-value ^c^
**Lineage 1** (Indo-Oceanic)	8.0	8.2	9.1	5.7	1.67 (0.46 to 6.04)	3.4 (-5.2 to 12.1)	0.565 *	8.3	1.1 (0.3 to 4.1)	0.8 (-9.7 to 11.2)	1 *
**Lineage 2** (East Asian *Beijing*)	5.6	5.6	6.3	3.8	1.71 (0.36 to 8.09)	2.5 (-4.7 to 9.8)	0.730 *	5.6	1.14 (0.23 to 5.55)	0.7 (-8.1 to 9.5)	1 *
**Lineage 3** (East African Indian)	1.0	1.0	1.4	0	-	1.4 (-1.8 to 4.6)	0.1 *	0	-	1.4 (-2.5 to 5.3)	1 *
**Lineage 4** (Euro-American)	51.8	51.5	**55.2**	41.5	1.74 (0.92 to 3.29)	13.7 (-2.1 to 29.5)	0.087	**30.5**	**2.81** (1.30 to 6.03)	**24.7** (6.4 to 43.0)	**0.008**
**Lineage 5** (*M*. *Africanum* West African 1)	25.1	25.0	**21**	**35.8**	**0.48** (0.24 to 0.94)	**-14.9** (-28.6 to -1.2)	**0.033**	41.7	**0.37** (0.17 to 0.8)	**-20.7** (-36.6 to -4.8)	**0.011**
**Lineage 6 (***M*. *Africanum* West African 2)	8.5	8.7	7	13.2	0.49 (0.18 to 1.36)	-6.2 (-15.1 to 2.7)	0.17	13.9	0.47 (0.15 to 1.43)	-6.9 (-17.1 to 3.3)	0.182
**Modern lineages (L2 + L3 + L4)**	58.3	58.2	**62.9**	**45.3**	**2.05** (1.09 to 3.87)	**17.7** (2.1 to 33.2)	**0.026**	**36.1**	**3.0** (1.43 to 6.31)	**26.8** (8.7 to 44.9)	**0.004**
**Ancestral lineages (L1 + L5 + L6)**	41.7	41.8	**37.1**	**54.7**	**0.49** (0.26 to 0.92)	**-17.7** (-33.2 to -2.1)		**63.9**	**0.33** (0.16 to 0.7)	**-26.8** (-44.9 to -8.7)	
**Other than *M*. *africanum* (L1 + L2 + L3 + L4)**	66.3	66.3	**72.0**	**50.9**	**2.48** (1.30 to 4.72)	**21.1** (6.2 to 36.0)	**0.006**	**44.4**	**3.22** (1.55 to 6.7)	**27.6** (10.3 to 44.9)	**0.002**
***M*. *africanum* (L5 + L6)**	33.7	33.7	**28**	**49.1**	**0.40** (0.21 to 0.77)	**-21.1** (-36.0 to -6.2)		**55.6**	**0.31** (0.15 to 0.65)	**-27.6** (-44.9 to -10.3)	

^**a**^ Based on sputum results in 196 patients, and culture results in 3 patients (for whom direct spoligotyping failed: 2 ‘Lineage 4’ strains and 1 ‘Lineage 5’ strain).

^**b**^ Direct spoligotyping (on sputa). L: Lineage.

^**c**^ p-values were calculated using the two-group proportion test (independent groups).

^*^ p-values were calculated using the Fisher Exact test (independent groups).

Excluding discrepant spoligotypes between direct and indirect spoligotype analysis, the association gained further statistical significance. The odds of detecting ancestral lineages in positive-cultures was 0.30 fold (95% CI (0.14 to 0.64); p = 0.0016) less in positive-cultures relative to negative cultures, especially Lineage 5 (OR = 0.37 95%CI (0.17 to 0.79); p = 0.010) (S1 Table). Modern lineages were inversely more represented in positive-culture specimens (OR = 3.31, 95%CI (1.57 to 6.99), p = 0.0016), especially Lineage 4 (OR = 3.01, 95%CI (1.4 to 6.51), p = 0.005) (S1 Table).

The prevalence of L1, L5, L6 tended to be higher among culture-negative specimens (respectively 8.3%, 41.7%, 13.9%; S1 Table) than in culture-positive specimens (7.4%, 20.7%, 6.7%; S1 Table). In contrast the prevalence of L2, L3, L4 tended to be lower among culture-negative specimens (5.6%, 0%, 30.5%) than in culture-positive specimens (6.7%, 1.5%, 57.0%; S1 Table). This justified the analysis in subgroup of ancestral and modern lineages. The distribution of sub-lineages (families) within Lineage 4 showed that LAM 10, LAM 9, LAM 1, T1, T2, Haarlem 1, Haarlem 2, Haarlem 3, X3 families were present in new TB patients in Cotonou. This distribution of Lineage 4 families did not differ significantly in culture-positive versus culture-negative sputa (S2 Table).

### Incubation time to culture positivity across lineages

Almost all positive cultures were positive within 8 weeks of incubation, while prolonged incubation only yielded one additional positive culture. This was a Lineage 5/ *M*. *africanum* West African 1 strain.

Among positive cultures, over half (5/9: 55.5%) of the Lineage 6/ *M*. *africanum* West African 2 cultures became positive between 6 to 8 weeks of incubation, whereas most of positive cultures from other lineages specimens were positive within 6 weeks: 10/11 (90.9%) for Lineage 1, 10/10 (100%) for Lineage 2, 2/2 (100%) for Lineage 3, 83/85 (97.6%) for Lineage 4 and 28/29 (95.6%) for Lineage 5.

Despite the prolonged incubation period, over a third of specimens from each *M*. *africanum* lineage remained culture negative (34.1% for Lineage 5 and 35.7% for Lineage 6), while for other lineages, none (Lineage 3) or fewer specimens (21.4% for Lineage 1, 16.7% for Lineage 2, 11.5% for Lineage 4) remained negative (Table 3). The sediment smear of the culture negative specimens from Lineage 1 and 2 had low AFB-grading or were negative whereas nearly all (14/15) the culture negative specimens from Lineage 5 had high smear grading (S3 Table).

**Table 3:** Time to culture positivity (on LJ medium) across lineages.

Lineages/Groups	Time to culture positivity ^a^			Culture-negative sputa^b^, n (%)	Total, n
	< 6 weeks, n	6–8 weeks, n	> 8–13 weeks, n		
**Lineage 1** (Indo-Oceanic)	**10**	1	0	3 (21.4)	14
**Lineage 2** (East Asian *Beijing*)	**10**	0	0	2 (16.7)	12
**Lineage 3** (East African Indian)	**2**	0	0	0 (0)	2
**Lineage 4** (Euro-American)	**83**	2	0	11 (11.5)	96
**Lineage 5** (*M*. *Africanum* West African 1)	**28**	0	**1**	**15 (34.1)**	44
**Lineage 6 (***M*. *Africanum* West African 2)	4	**5**	**0**	**5 (35.7)**	14

^**a**^ Lineages were determined using indirect spoligotyping (culture isolates)

^**b**^ Direct spoligotyping (on sputa) used. Sputa with contaminated culture were not included.

## Discussion

Our results show that indirect spoligotyping provided spoligotype profiles for all 146 culture-positive specimens (73.4%), while direct spoligotyping provided spoligotyping profiles for 50 more sputa (+ 25.1% of all 199 specimens, 95% CI (18.1% to 32.1%)) that would not otherwise be genotyped in the absence of an isolate. Direct spoligotyping on sputum after semi-automated DNA extraction using Maxwell DNA tissue purification kit, has a high sensitivity (98.5% (196/199)) to detect MTBc genotypes. The 98.5% (196/199) overall availability of spoligotype profiles in our study is higher than the 90.9% (159/175) found on smear-positive sputa by Goyal et *al*. in Ghana (p = 0.001) [18] and the 49.1% (28/57) found by Heyderman et *al*. in Zimbabwe [17]. This could be explained by the variability of methods used for DNA extraction from sputa and/or the variability in PCR reagents mix. The overall availability of spoligotype profiles on sputa in our study (98.5%) is also higher than the 77.7% (41/53) found by Suresh et *al*. and 90.5% (19/21) by Zanden et *al*. on smears [19,28], which likely have less mycobacterial DNA than a 200 μL sputum sample.

The fact that- within mixed infections- ancestral lineages are found with direct spoligotyping on sputum, suggests that the load of ancestral lineage bacilli *in vivo* exceeds the load of the modern lineage bacilli, with subsequent out-competition in culture by the latter. Sarkar et *al*. also found that Lineage 4 grows more rapidly (in liquid medium) than other lineages including Lineage 1, an ancestral lineage [29]. Moreover, Gehre et *al*. found that Lineage 6, another ancestral lineage, grows more slowly than MTBc lineages other than *M*. *africanum* in liquid medium [12].

Sputum provided the most representative population distribution of lineages of the MTBc in new TB patients in Cotonou, with more TB due to ancestral lineages, including *M*. *africanum*. This distribution did not alter when the three isolates which sputum failed direct spoligotyping were added (two from Lineage 4 and one from Lineage 5; Table 2). The ‘most true’ distribution is the one combining profiles obtained directly from sputum, complemented by profiles on isolates from failed direct spoligotyping, and includes: 8.0% (16/199) for Lineage 1, 5.6% (11/199) for Lineage 2, 1% (2/199) for Lineage 3, 51.8% (103/199) for Lineage 4, 25.1% (50/199) for Lineage 5, 8.5% (17/199) for Lineage 6, or 41.7% for ancestral lineages, and 33.7% for *M*. *africanum* (Table 2). This distribution would have been different if smear-negative specimens were also genotyped, as it had been previously reported that *M*. *africanum* is more likely to be found in lower grade smear-positive specimens [30], and Lineage 6 is associated with HIV infection [31], which is in turn associated with smear-negativity [32–34].

The comparison of the distribution of MTBc lineages in a similar population, also consisting of consecutive smear-positive new pulmonary TB patients aged at least 15 years old of Cotonou in year 2005–2006 on cultured isolates [9,35], to the one obtained in our study indirectly on cultured isolates from similar patients in Cotonou 10 years later, showed that the previous prevalence of Lineage 1 (7.7%), Lineage 2 (10.3%), Lineage 3 (0%), Lineage 6 (6.2%) are similar to our findings in this study (respectively: 7.5%, 6.8%, 1.4% and 6.2%). Yet the prevalence of Lineage 4 (42.3% in year 2005–2006) has increased to 58.2% (difference: +15.9%), and Lineage 5 prevalence (30.9% in year 2005–2006) has decreased to 19.9% (difference: -11.0%). While we demonstrate that the present L5 prevalence of 19.9% on indirect genotyping is an underestimate, even the present ‘true’ L5 prevalence of 25.1% on direct genotyping would constitute a decline from the L5 prevalence of 30.9% on indirect genotyping in 2005–2006. Other authors also reported a decrease of *M*. *africanum* [8,9].

Our results show that rates of culture isolation from smear-positive pulmonary TB patients are lower for Lineages 5 and 6 of the MTBc, despite prolonged incubation of cultures for 90 days [13]. Extending the incubation time beyond 6 weeks enhanced isolation of Lineage 6 (between 6–8 weeks) yet did not further augment the isolation rate. Ancestral lineages, especially Lineage5/*M*. *africanum* West African 1 are ‘difficult-growers’ in culture relative to modern lineages, such as Lineage 4. The decreased odds of ancestral lineages to grow in culture could partly be due to culture procedures (culture medium or decontamination method) that were originally developed for modern lineages prevalent in Europe. Ofori-Anyinam et *al*. reported that Lineage 6 as compared to Lineage 4, is more adapted to microaerobic growth [14] which may be the reason for its impaired growth on solid media such as LJ used in this study. Furthermore Gehre et *al*. found that Lineage 6 has mutations in genes that lead to its attenuated growth in vitro[12]. Such genetic analyses need to be conducted on Lineage 5 in order to understand the reasons for its difficult growth in vitro. Further studies should also be conducted on other lineages to find out the genetic basis of their in vitro growth pattern. To the best of our knowledge, this is the first demonstration that ancestral lineages are underrepresented in positive cultures. Direct spoligotyping is thus more appropriate for unbiased determination of MTBc population structure in settings where ancestral lineages, including *M*. *africanum*, are common.

The implications of our findings also affect MTBc population structures generated with different typing methods, including whole genome sequencing. Such studies tend to be culture-based, given the ongoing limitations of sequencing entire MTBc genomes directly from clinical material. While direct genome sequencing is technically feasible given sufficient coverage, in practice the associated costs are prohibitive. Studies to date have shown limited coverage, precluding SNP cut-offs for molecular epidemiological studies [36]. Optimized methods to sequence genomes directly from clinical material are thus urgently needed.

One strength of this study is the prolonged incubation time, to maximize the yield of *M*. *africanum* in culture. Other strengths include the paired design for the comparison of direct spoligotyping versus indirect spoligotyping and the inclusion of multiple controls and blinding of operators, and the fact that the study was conducted in a setting where *M*. *africanum* is prevalent. A limitation is that only LJ medium was used, and we do not know whether other medium, such as liquid medium (known to enable the growth of more non-tuberculous mycobacteria) may also favor the growth of ancestral MTBc lineages. This study was conducted only on fresh unshipped acid-fast bacilli positive sputa from new TB patients. Culture positivity may be worse if sputa had to be shipped from peripheral laboratories to a reference or central laboratory where spoligotyping can be done. Another limitation is that the number of specimens with Lineages 1, 2, 3, 6 among culture negative specimens under-powered the estimation of any difference in the prevalence of these individual lineage among culture-negative versus culture-positive specimens. So, although no evidence of such difference in prevalence among culture-negative versus –positive specimens was found in Lineages 1, 2, 3, 6 in the present study, such difference could be tested for in settings with higher prevalence of these lineages.

In conclusion, ancestral lineages especially *M*. *africanum* West African 1 (Lineage 5), are less likely to grow in culture, unlike modern lineages especially *M*. *tuberculosis* Euro-American (Lineage 4). Direct spoligotyping on sputum is effective, and saves effort and time compared to indirect spoligotyping of cultures. It has an important gain in sensitivity, especially for ancestral lineages that may not yield a positive culture, allowing a more precise unbiased determination of the population structure of the MTBc. It can also be used for specimens from patients under TB treatment and other specimens in which culture may be negative or contaminated. While differences in culture isolation technique and reliance on indirect spoligotyping may partially account for the reduction in the prevalence of *M*. *africanum* observed in several West African countries [8,9], comparison of our findings with the genotyping study from Cotonou 10 years ago suggests that the decline in *M*. *africanum* is not explained by the lower sensitivity of culture isolation. The potential decline of *M*. *africanum* lineages will be addressed in more depth in a larger ongoing study on the population structure of the *M*. *tuberculosis* complex in Benin, in which direct genotyping will be applied, given the findings presented in this manuscript. Further studies must be conducted to investigate whether culture procedures (medium, decontamination) can be optimized for growth of ancestral lineages. Additional studies should address the frequency and role, if any, of a mixed infection between an ancestral- and modern lineage in the faster spread of modern lineages [4] and disappearance of ancestral lineages [8,9].

## Supporting information

S1 Table Effect of prior culture on spoligotyping analysis for MTBc lineage detection: Sensitivity analysis excluding all (spoligotype and sub(lineage) level) discrepancies (between direct versus indirect spoligotyping). Direct spoligotyping is used for all sputa in this comparison.
(DOCX)

S2 Table Distribution of sub-lineages (families) within Lineage 4 depending on culture result. (DOCX)

S3 Table AFB-microscopy of sediments in positive and negative cultures across lineages of the MTBc. (DOCX)
