# Supplementary material for: Genotypic characterization directly applied to sputum improves the detection of Mycobacterium africanum West African 1, under-represented in positive cultures
Source: PLoS Negl Trop Dis. 2017 Sep 1;11(9):e0005900. doi: 10.1371/journal.pntd.0005900 (PMC5599059; doi:10.1371/journal.pntd.0005900)
Supplement: S1 Table — Direct spoligotyping is used for all sputa in this comparison. (DOCX) [file pntd.0005900.s001.docx]

**S1 Table. Effect of prior culture on spoligotyping analysis for MTBc lineage detection: Sensitivity analysis excluding all (spoligotype and sub(lineage) level) discrepancies (between direct versus indirect spoligotyping).** Direct spoligotyping is used for all sputa in this comparison.

| **Lineages/Groups** | **Culture positive specimens ^a, b^ % (n=135)** | **Culture Negative & Contaminated specimens** | | | | **Culture Negative** specimens (**only**) | | | |
| --- | --- | --- | --- | --- | --- | --- | --- | --- | --- |
|  |  | **% (n=53)** | **Odds ratio (Odd_Pos cult_ / Odd_Neg & Cont cult_**) with **95% CI** | **Difference (P_Pos cult_ - P_Neg & Cont cult_)** with **95% CI** | **p-value** ^c^ | **% (n=36)** | **Odds ratio ( Odd_Pos cult_ / Odd_Neg cult_**) with **95% CI** | **Difference (P_Pos Cult_ - P_Neg cult_)** with **95% CI** | **p-value ^c^** |
| **Lineage 1** (Indo-Oceanic) | 7.4 | 5.7 | 1.33 (0.35 to 5.04) | 1.7 (-6.3 to 9.8) | 1 * | 8.3 | 0.88 (0.23 to 3.39) | -0.9 (-10.7 to 8.8) | 0.738 * |
| **Lineage 2** (East Asian *Beijing*) | 6.7 | 3.8 | 1.82 (0.39 to 8.6) | 2.9 (-4.6 to 10.4) | 0.731 * | 5.6 | 1.21 (0.25 to 5.9) | -1.1 (-7.9 to 10.1) | 1 * |
| **Lineage 3** (East African Indian) | 1.5 | 00 | - | 01.5 (-1.8 to 4.8) | 1 * | 0 | - | 1.5 (-2.5 to 5.4) | 1 * |
| **Lineage 4** (Euro-American) | **57.0** | 41.5 | 1.87 (0.98 to 3.55) | 15.5 (-0.4 to 0.3) | 0.055 | **30.5** | **3.01** (1.4 to 6.51) | **26.5** (8.0 to 44.9) | **0.005** |
| **Lineage 5** (*M. Africanum* West African 1) | **20.7** | **35.8** | **0.47** (0.23 to 0.94) | **-15.1** (-28.9 to -1.3) | **0.031** | **41.7** | **0.37** (0.17 to 0.79) | **-20.9** (-36.9 to -4.9) | **0.010** |
| **Lineage 6 (***M. Africanum* West African 2) | 06.7 | 13.2 | 0.47 (0.17 to 1.31) | -6.5 (-15.4 to 2.3) | 0.148 | 13.9 | 0.44 (0.14 to 1.38) | -7.2 (-17.3 to 2.9) | 0.160 |
| **Modern lineages (L2 + L3 + L4)** | **65.2** | **45.3** | **2.26** (1.19 to 4.3) | **19.9** (4.3 to 35.5) | **0.012** | **36.1** | **3.31** (1.57 to 6.99) | **29.1** (10.9 to 47.2) | **0.0016** |
| **Ancestral lineages (L1 + L5 + L6)** | **34.8** | **54.7** | **0.44** (0.23 to 0.84) | **-19.9** (-35.5 to -4.3) |  | **63.9** | **0.30** (0.14 to 0.64) | **-29.1** (-47.2 to -10.9) |  |
| **Other than *M. africanum* (L1 + L2 + L3 + L4)** | **72.6** | **50.9** | **2.55** (1.33 to 4.89) | **21.6** (6.6 to 36.7) | **0.005** | **44.4** | **3.31 (1.58 to 6.93)** | **28.1** (10.8 to 45.5) | **0.0015** |
| ***M. africanum* (L5 + L6)** | **27.4** | **49.1** | **0.39** (0.2 to 0.75) | **-21.6** (-36.7 to -6.6) |  | **55.6** | **0.30** (0.14 to 0.63) | **-28.2** (-45.5 to -10.8) |  |

**^a^** All (8) discrepancies (between direct versus indirect spoligotyping) were excluded. **^b^** Direct spoligotyping (on sputa) used. **^c^** p-values were calculated using the two-group proportion test (independent groups). **^*^** p-values were calculated using the Fisher Exact test (independent groups).
