# Supplementary material for: Genotypic characterization directly applied to sputum improves the detection of Mycobacterium africanum West African 1, under-represented in positive cultures
Source: PLoS Negl Trop Dis. 2017 Sep 1;11(9):e0005900. doi: 10.1371/journal.pntd.0005900 (PMC5599059; doi:10.1371/journal.pntd.0005900)
Supplement: S2 Table — (DOCX) [file pntd.0005900.s002.docx]

| **Families (sublineages) within L4** | **All (L4) sputa ^a^ n=101** | **All (L4) isolates ^b^ n=85** | **Culture positive sputa ^a^ n=79** | **Culture Negative & Contaminated sputa ^d^** | | | **Culture Negative** sputa (**only**) ^d^ | | |
| --- | --- | --- | --- | --- | --- | --- | --- | --- | --- |
|  |  |  |  | **n=22** | **% Difference (P_Pos cult_ - P_Neg & Cont cult_)** with **95% CI** | **p-value** **^c^** | **n=11** | **% Difference (P_Pos cult_ - P_Neg cult_)** with **95% CI** | **p-value** **^c^** |
| **LAM 1** | 2 | 2 | 2 | 0 | 2.5 (-4.1 to 9.1) | 1 | 0 | 2.5 (-6.8 to 11.9) | 1 |
| **LAM 9** | 4 | 4 | 4 | 0 | 5.1 (-4.2 to 14.3) | 0.574 | 0 | 5.1 (-8 to 1.8) | 1 |
| **LAM 10** | 68 (67.3%) | 55 (64.7%) | 52 (65.8%) | 16 (72.7%) | -6.9 (-29.2 to 15.4) | 0.615 | 10 (90.9%) | -25.1 (-54.4 to 4.3) | 0.162 |
| **T1** | 15 (14.8%) | 13 (15.3%) | 12 (15.2%) | 3 (13.6%) | 1.6 (-15.3 to 18.4) | 1 | 1 (9.1%) | 6.1 (16.2 to 28.4) | 1 |
| **T2** | 1 | 1 | 1 | 0 | 1.3 (-3.4 to 6) | 1 | 0 | 1.3 (-5.4 to 7.9) | 1 |
| **Haarlem 1** | 4 | 6 | 4 | 0 | 5.1 (-4.2 to 14.3) | 0.574 | 0 | 5.1 (-8.0 to 18.1) | 1 |
| **Haarlem 2** | 1 | 1 | 1 | 0 | 1.3 (-3.4 to 6) | 1 | 0 | 1.3 (-5.4 to 7.9) | 1 |
| **Haarlem 3** | 4 | 2 | 2 | 2 | -6.6 (-15.8 to 2.7) | 0.206 | 0 | 2.5 (-6.8 to 11.9) | 1 |
| **X3** | 2 | 1 | 1 | 1 | -3.3 (-9.9 to 3.3) | 0.390 | 0 | 1.3 (-5.4 to 7.9) | 1 |

**S2 Table. Distribution of sub-lineages (families) within Lineage 4 depending on culture result**

**^a^** Direct spoligotyping (on sputa).  **^b^** Indirect spoligotyping (on isolates). L: Lineage

**^c^** p-values were calculated using the Fisher Exact test (independent groups). **^d^** Same even when discrepancies were excluded.
