# Supplementary material for: Genotypic characterization directly applied to sputum improves the detection of Mycobacterium africanum West African 1, under-represented in positive cultures
Source: PLoS Negl Trop Dis. 2017 Sep 1;11(9):e0005900. doi: 10.1371/journal.pntd.0005900 (PMC5599059; doi:10.1371/journal.pntd.0005900)
Supplement: S3 Table — (DOCX) [file pntd.0005900.s003.docx]

**S3 Table. AFB-microscopy of sediments in positive and negative cultures across lineages of the MTBc**

| **Lineages/Groups** | **AFB-microscopy in sediment** | | | | | | | | | |
| --- | --- | --- | --- | --- | --- | --- | --- | --- | --- | --- |
|  | **Culture positive** specimens ^a^ | | | | | **Culture negative** specimens ^b^ | | | | |
|  | **+++** | **++** | **+** | **Scanty** | **Negative** | **+++** | **++** | **+** | **Scanty** | **Negative** |
| **Lineage 1** (Indo-Oceanic) | 4 | 5 | 3 | - | - | - | **-** | **-** | **2** | **1** |
| **Lineage 2** (East Asian *Beijing*) | 3 | 5 | 2 | - | - | - | - | 1 | 1 | - |
| **Lineage 3** (East African Indian) |  | 2 | - | - | - | - | - | - | - | - |
| **Lineage 4** (Euro-American) | 28 | 36 | 11 | 4 | 3 | 1 | 5 | 4 | 1 | - |
| **Lineage 5** (*M. Africanum* West African 1) | 5 | 19 | 5 | 1 | 1 | **11** | **3** | - | - | 1 |
| **Lineage 6 (***M. Africanum* West African 2) | 1 | 7 | 1 | - | - | 1 | 2 | 1 | 1 | - |

**^a^** Lineages were determined using indirect spoligotyping (culture isolates)

**^b^** Direct spoligotyping (on sputa) used. Sputa with contaminated culture were not included.

- means 0 (zero) specimen.
